# Comparison of inspiratory and expiratory lung and lobe volumes among supine, standing, and sitting positions using conventional and upright CT

**DOI:** 10.1038/s41598-020-73240-8

**Published:** 2020-10-01

**Authors:** Yoshitake Yamada, Minoru Yamada, Shotaro Chubachi, Yoichi Yokoyama, Shiho Matsuoka, Akiko Tanabe, Yuki Niijima, Mitsuru Murata, Koichi Fukunaga, Masahiro Jinzaki

**Affiliations:** 1 Department of Radiology, Keio University School of Medicine, 35 Shinanomachi, Shinjuku-ku, Tokyo, 160-8582 Japan; 2 Division of Pulmonary Medicine, Department of Medicine, Keio University School of Medicine, 35 Shinanomachi, Shinjuku-ku, Tokyo, 160-8582 Japan; 3 Department of Clinical Laboratory, Keio University Hospital, 35 Shinanomachi, Shinjuku-ku, Tokyo, 160-8582 Japan; 4 Office of Radiation Technology, Keio University Hospital, 35 Shinanomachi, Shinjuku-ku, Tokyo, 160-8582 Japan; 5 Department of Laboratory Medicine, Keio University School of Medicine, 35 Shinanomachi, Shinjuku-ku, Tokyo, 160-8582 Japan

**Keywords:** Anatomy, Medical research, Medical imaging

## Abstract

Currently, no clinical studies have compared the inspiratory and expiratory volumes of unilateral lung or of each lobe among supine, standing, and sitting positions. In this prospective study, 100 asymptomatic volunteers underwent both low-radiation-dose conventional (supine position, with arms raised) and upright computed tomography (CT) (standing and sitting positions, with arms down) during inspiration and expiration breath-holds and pulmonary function test (PFT) on the same day. We compared the inspiratory/expiratory lung/lobe volumes on CT in the three positions. The inspiratory and expiratory bilateral upper and lower lobe and lung volumes were significantly higher in the standing/sitting positions than in the supine position (5.3–14.7% increases, all P < 0.001). However, the inspiratory right middle lobe volume remained similar in the three positions (all P > 0.15); the expiratory right middle lobe volume was significantly lower in the standing/sitting positions (16.3/14.1% decrease) than in the supine position (both P < 0.0001). The Pearson’s correlation coefficients (*r*) used to compare the total lung volumes on inspiratory CT in the supine/standing/sitting positions and the total lung capacity on PFT were 0.83/0.93/0.95, respectively. The *r* values comparing the total lung volumes on expiratory CT in the supine/standing/sitting positions and the functional residual capacity on PFT were 0.83/0.85/0.82, respectively. The *r* values comparing the total lung volume changes from expiration to inspiration on CT in the supine/standing/sitting positions and the inspiratory capacity on PFT were 0.53/0.62/0.65, respectively. The study results could impact preoperative CT volumetry of the lung in lung cancer patients (before lobectomy) for the prediction of postoperative residual pulmonary function, and could be used as the basis for elucidating undetermined pathological mechanisms. Furthermore, in addition to morphological evaluation of the chest, inspiratory and expiratory upright CT may be used as an alternative tool to predict lung volumes such as total lung capacity, functional residual capacity, and inspiratory capacity in situation in which PFT cannot be performed such as during an infectious disease pandemic, with relatively more accurate predictability compared with conventional supine CT.

## Introduction

Although humans spend most of their daytime in an upright position, the detailed human anatomy of the lung in the standing or sitting position remains unclear. Chest radiography can be performed in the upright position^1^; however, chest radiography provides a two-dimensional image and cannot allow for the accurate measurement of the lung or lobe volume.

Recently, a 320-detector-row upright computed tomography (CT) scanner has been developed to evaluate human anatomy in the upright position three-dimensionally, and can be helpful to clarify the effects of gravity on the entire human body^2^, although there are already several studies describing the effect of gravity and posture on the lungs^3–5^. This upright CT scanner allows for the acquisition of isotropic volume data (isotropic 0.5-mm voxel size) of the whole chest within approximately 5 seconds^6^.

It has been reported that the total lung capacity measured by pulmonary function test (PFT) in the standing or sitting position is higher than that determined in the supine position^7,8^; however, unilateral lung volume or each lung lobe volume cannot be measured by PFT. Conversely, CT images can provide the volumes of the unilateral lung and each lung lobe^9–12^ and reportedly enables more accurate predictions of postoperative residual pulmonary function in patients with lung cancer than does the segment-counting method, which is based solely on the number of remaining pulmonary segments^9–11^. Upright CT provides images of daily-life postures (both standing and sitting positions) and possibly enables a more accurate prediction of postoperative residual pulmonary function, before lobectomy, than does conventional CT (supine position)^6^.

A previous, preliminary study compared lung and lobe volumes between supine and standing positions using conventional and upright CT; however, supine CT was performed with the subjects’ arms down, while conventional chest CT in the supine position is usually performed with the subject’s arms raised^6^. In addition, the previous study evaluated only inspiratory lung/lobe volumes in the standing position^6^, but not expiratory lung/lobe volume or lung/lobe volume in the sitting position. To the best of our knowledge, no clinical studies to date have accurately compared both the inspiratory and expiratory lung/lobe volumes in the supine (with the subject’s arms raised), standing, and sitting positions. We hypothesized that the inspiratory/expiratory volume change rates of each lobe of the lung between supine and upright (standing and sitting) positions would be different, because the direction of gravity relative to the chest differs between supine and upright positions.

The purpose of this study was thus to compare inspiratory and expiratory lung/lobe volumes on CT among supine, standing, and sitting positions.

## Methods

### Study population

This prospective study was approved by the Keio University School of Medicine Ethics Committee (No. 20160384). Written informed consent was obtained from all participants (UMIN Clinical Trials Registry [UMIN-CTR]: UMIN000026586). All methods were performed in accordance with the relevant guidelines and regulations. From June 2017 to August 2018, 100 asymptomatic volunteers from a volunteer recruitment company were enrolled in this study. To evaluate normal whole-body anatomy, volunteers with a history of smoking, diabetes, hypertension, dyslipidemia, and dysuria; those who had any type of symptom; had undergone any surgery; or were currently undergoing treatment and pregnant or possibly pregnant volunteers were excluded from the study.

### CT imaging protocol

**Figure 1:**
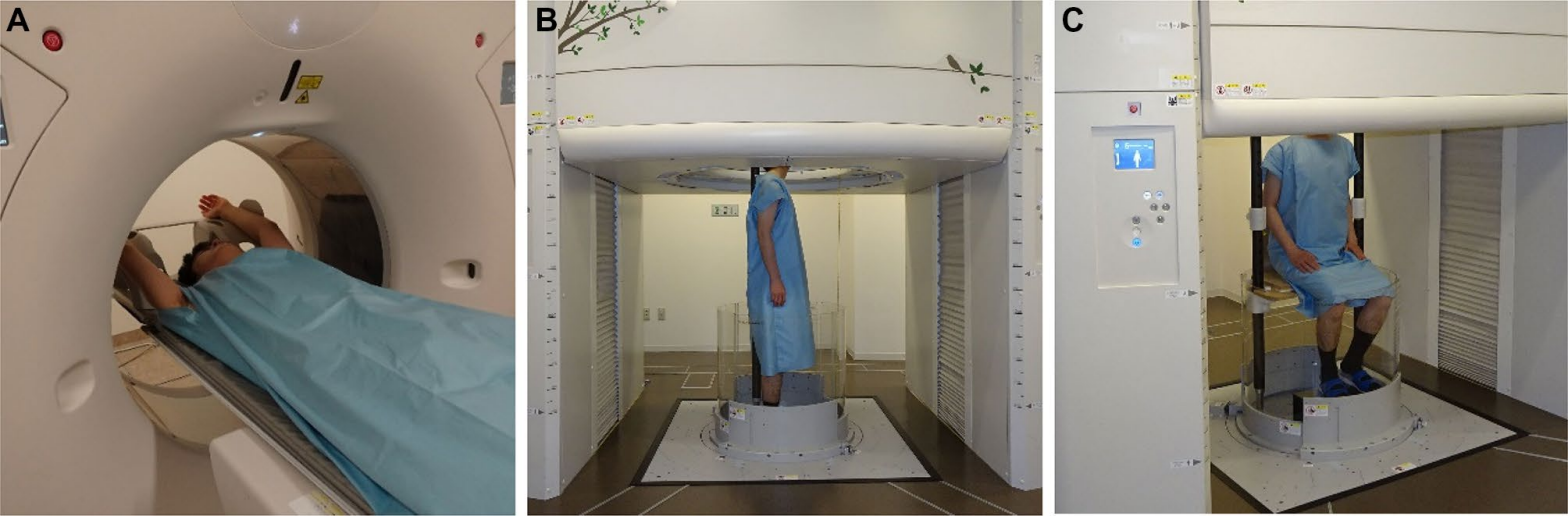
Conventional CT examination in the supine position (**A**), upright CT examination in the standing position (**B**) and upright CT examination in the sitting position (**C**). Conventional CT in the supine position (**A**) was performed with the subject’s arms raised during both deep-inspiration breath-hold and expiration breath-hold. Upright CT examinations in the standing position (**B**) and sitting position (**C**) were performed with the subject’s arms down during both deep-inspiration breath-hold and expiration breath-hold. For safety during upright CT scanning, an acrylic wall encircling the body was added to prevent falls, with a pinch prevention mechanism and contact interlock control mechanism. Furthermore, to stabilize patients while standing, a back-support pole, 2.3-m long and made of carbon, was included; it was mounted between the floor and the top of the system (**B**). The mounting position could be adjusted based on the subject's bodily dimensions or the scan conditions. To support patients who are frail or elderly, a Velcro band can be attached to the pole and loosely wrapped around a patient's body. In this study, the subjects were healthy volunteers; thus, we did not use the Velcro band.

All participants underwent both conventional body trunk low-radiation-dose CT in the supine position with arms raised (Fig. 1A) performed using a 320-detector-row CT (Aquilion ONE, Canon Medical Systems, Otawara, Japan) and upright body trunk low-radiation-dose CT in standing (Fig. 1B) and sitting positions with arms down (Fig. 1C) performed using a 320-detector-row upright CT (prototype TSX-401R, Canon Medical Systems)^2,6^ on the same day. Subjects were scanned in the three positions in breath-hold both at the end-inspiration, near total lung capacity on PFT, and at the end-tidal expiration, near functional residual capacity on PFT, as described in a previous study^13^. Additionally, head and neck scans in the supine position and head scan in the sitting position were obtained for these volunteers; however, only chest-related data were analyzed in this study.

For the body trunk, all low-radiation-dose CT examinations were unenhanced and were performed with automatic exposure control using a noise index of 24 for a slice thickness of 5 mm (tube current range, 10–350 mA). Other scanning parameters were also the same for supine, standing, and sitting CT scans: peak tube voltage, 100 kVp; rotation speed, 0.5 s; slice collimation, 0.5 mm × 80; and pitch factor, 0.813. The series of contiguous 0.5-mm-thick images was reconstructed with Adaptive Iterative Dose Reduction 3D (Canon Medical Systems)^14^. The sum of the CT dose index volume for inspiratory and expiratory CT in the supine, standing, and sitting CT positions was 14.97 ± 3.43 mGy (supine CT, 4.39 ± 1.01 mGy; standing CT, 5.39 ± 1.28 mGy; sitting CT, 5.19 ± 1.39 mGy).

### Pulmonary function test

All participants underwent PFTs on the same day of CT examinations (within two hours of CT examinations). The PFT was performed in a stable condition, with the subject in a sitting position, using a spirometer (Chestac-8900, Chest M.I., Tokyo, Japan) in accordance with ATS/European Respiratory Society recommendations^15,16^. The residual volume and total lung capacity were measured using the multibreath helium dilution method. Predicted values of spirometric measurements were derived from the guidelines of the Japanese Respiratory Society^17^.

### Lung and lobe volume measurements using CT

**Figure 2:**
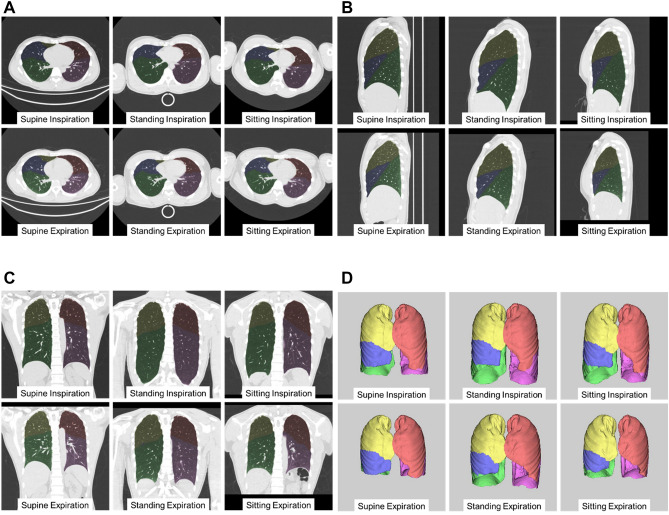
Representative segmentation and inspiratory/expiratory lung/lobe volume measurement in a 39-year-old male subject: axial (**A**), sagittal (**B**), and coronal (**C**) images, and volume rendering images (**D**) acquired in the supine, standing, and sitting positions. Yellow is the right upper lobe, blue is the right middle lobe, green is the right lower lobe, pink is the left upper lobe, and purple is the left lower lobe.

The first lung and lobe volume measurements on CT of all 100 volunteers in each position were performed by a chest radiologist with 14 years of experience (Y.Y.), using a commercially available workstation (Synapse Vincent, Fuji Film Co., Ltd., Tokyo, Japan). This workstation incorporated a lobar computer-aided diagnosis (CAD) system that was demonstrated to measure lobar volumes precisely in a previous study^18^. This system automatically extracted right and left lungs, recognized lobar bronchi, and determined the locations of fissures (Fig. 2)^6,19^. The chest radiologist verified the CAD-determined results of segmentation and made manual corrections by delineating fissures when the CAD system failed to identify them properly, as described in a previous study^6,19^. Approximately one-third of the segmentations required minor manual correction (less than 10% change of the volumes in all the corrected cases). A second measurement of the first 20 volunteers was performed by the same radiologist to assess intraobserver agreement, 1 month after the first assessment. To assess interobserver agreement, lung volumes of the first 20 volunteers were measured by a different general radiologist with 5 years of experience (Y.Y.).

All measurements were performed in a randomized and blinded manner. During all measurements, the radiologists were also blinded to participants’ characteristics and results of the PFT. The proportional volumes of each lung and each lobe relative to the total lung volume, lung and lobe volume changes from expiration to inspiration on CT, and the ratio of inspiratory lung/lobe volume to expiratory lung/lobe volume were also calculated.

### Statistical analysis

Data are presented as mean ± standard deviation. Paired t-tests were performed to analyze differences in the volumes of the total lungs, right lung, left lung, and each lobe among supine, standing, and sitting positions; the differences in the proportional volumes of each lung and each lobe relative to the total lung volume among the three positions; the differences in volume changes from expiration to inspiration among the three positions; and the differences in the ratio of inspiratory lung/lobe volume to expiratory lung/lobe volume among the three positions. Bonferroni correction was used for multiple comparisons. The difference in age between women and men was assessed using Student’s *t*-test. Interobserver and intraobserver agreements were evaluated by measuring intraclass-correlation coefficients. The correlations between the volumes on CT in each position and the measurements on PFT (total lung capacity, functional residual capacity, and inspiratory capacity) were also evaluated by measuring intraclass-correlation coefficients. The associations between the volumes on CT in each position and the measurements on PFT (total lung capacity, functional residual capacity, and inspiratory capacity) were evaluated by Pearson’s correlation coefficient test. The significance level for all tests was 5% (two-sided). All data were analyzed using a commercially available software program (JMP version 12; SAS Institute Inc, Cary, NC, USA).

## Results

### Participant characteristics

The clinical characteristics of all the participants (n = 100) is shown in Table 1. No significant difference was found in age between women and men (48.0 ± 11.9 years vs. 44.9 ± 10.0 years, *P* = 0.210).

**Table 1:** Characteristics of the study population (100 volunteers).

Demographic variables	Value	
	Mean ± SD or n	Range
Age (years)	47.1 ± 11.4	30‒79
Sex (female/male) (n)	69/31	
Height (cm)	161.2 ± 9.0	141.4‒187.5
Weight (kg)	57.6 ± 12.1	37.8‒106.8
Body mass index (kg/m^2^)	22.1 ± 3.6	15.7‒33.7
Pulmonary function test		
VC (L)	3.63 ± 0.91	1.79‒6.13
FVC (L)	3.56 ± 0.91	1.79‒5.98
VC (% predicted)	105.4 ± 10.4	81.6‒138.9
FEV_1_ (L)	2.86 ± 0.74	1.37‒4.61
FEV_1_ (% predicted)	101.9 ± 12.2	71.1‒133.5
FEV_1_/FVC (%)	80.6 ± 7.5	55.5‒96.0
Tidal volume (L)	0.57 ± 0.17	0.29‒1.06
Residual volume (L)	1.51 ± 0.38	0.80‒2.92
Residual volume (% predicted)	126.3 ± 32.2	48.9‒212.6
Functional residual capacity (L)	2.91 ± 0.70	1.36‒4.86
Functional residual capacity (% predicted)	108.2 ± 18.5	62.5‒156.3
Total lung capacity (L)	5.13 ± 1.17	2.65‒8.22
Total lung capacity (% predicted)	111.4 ± 13.1	77.6‒157.0
DL_CO_ (mL/min/Torr)	22.4 ± 5.7	10.6‒38.2

SD, standard deviation; FEV_1_, forced expiratory volume in 1 s; FVC, forced vital capacity; VC, vital capacity; DL_CO_, diffusing capacity of the lung for carbon monoxide.

### Lung and lobe volumes on CT in supine, standing, and sitting positions

The inspiratory and expiratory bilateral upper and lower lobe and bilateral lung volumes were significantly higher in the standing and sitting positions than in the supine position (5.3–14.7% increases, all *P* < 0.001) (Table 2). However, no significant differences were found in the inspiratory right middle lobe volume among the three positions (all *P* > 0.15); the expiratory right middle lobe volume was significantly lower in standing (16.3% decrease) and sitting positions (14.1% decrease) than in the supine position (all *P* < 0.0001) (Table 2). No significant differences were found in the average inspiratory volumes of the total lung, right lung, left lung, and all lobes between standing and sitting positions (Table 2).

**Table 2:** Inspiratory and expiratory lung and lobe volumes as determined using CT in the supine position with arms raised, standing position with arms down, and sitting position with arms down (100 volunteers).

	Lung and lobe volumes on CT Average ± SD (Range)			*P* value			Average percent increase/decrease in volume in standing position compared to that in supine position (%)	Average percent increase/decrease in volume in sitting position compared to that in supine position (%)	Average percent increase/decrease in volume in sitting position compared to that in standing position (%)
	Supine (mL)	Standing (mL)	Sitting (mL)	Supine vs. standing	Supine vs. sitting	Standing vs. sitting			
Total (Bilateral) lung (Inspiratory)	4286.2 ± 1091.7 (2404.5‒7216.3)	4682.6 ± 1055.4 (2726.9‒7481.2)	4713.5 ± 1112.3 (2704.0‒7477.6)	< 0.0001	< 0.0001	0.3134	+ 9.2	+ 10.0	+ 0.7
Right lung (Inspiratory)	2286.4 ± 554.8 (1282.4‒3724.6)	2481.5 ± 540.4 (1550.8‒3956.4)	2508.4 ± 576.2 (1596.7‒3982.9)	< 0.0001	< 0.0001	0.1026	+ 8.5	+ 9.7	+ 1.1
Right upper lobe (Inspiratory)	786.1 ± 196.9 (383.3‒1289.2)	847.9 ± 198.0 (451.3‒1335.4)	852.8 ± 204.5 (435.4‒1382.4)	< 0.0001	< 0.0001	0.2484	+ 7.9	+ 8.5	+ 0.6
Right middle lobe (Inspiratory)	383.0 ± 111.6 (189.1‒747.6)	382.5 ± 106.6 (173.7‒670.8)	386.6 ± 112.9 (172.3‒678.8)	0.8896	0.3212	0.1588	− 0.1	+ 0.9	+ 1.1
Right lower lobe (Inspiratory)	1117.2 ± 313.4 (547.2‒1905.8)	1251.1 ± 303.9 (637.8‒2218.5)	1269.0 ± 322.7 (646.4‒2108.4)	< 0.0001	< 0.0001	0.0777	+ 12.0	+ 13.6	+ 1.4
Left lung (Inspiratory)	1999.9 ± 543.8 (1045.5‒3491.8)	2201.1 ± 521.4 (1100.7‒3524.8)	2205.1 ± 542.8 (1084.9‒3494.7)	< 0.0001	< 0.0001	0.7876	+ 10.1	+ 10.3	+ 0.2
Left upper lobe (Inspiratory)	1009.3 ± 250.2 (540.9‒1692.9)	1073.9 ± 243.0 (633.9‒1676.2)	1078.3 ± 251.8 (649.2‒1711.9)	< 0.0001	< 0.0001	0.4176	+ 6.4	+ 6.8	+ 0.4
Left lower lobe (Inspiratory)	990.5 ± 319.6 (382.5‒1798.8)	1127.3 ± 309.6 (418.0‒1905.8)	1126.8 ± 322.2 (413.3‒1913.2)	< 0.0001	< 0.0001	0.9621	+ 13.8	+ 13.8	− 0.0
Total (Bilateral) lung (Expiratory)	2688.0 ± 683.5 (1442.6‒4541.0)	2859.8 ± 760.0 (1388.4‒4937.8)	2943.6 ± 778.9 (1396.9‒5589.4)	< 0.0001	< 0.0001	0.0347	+ 6.4	+ 9.5	+ 2.9
Right lung (Expiratory)	1460.6 ± 356.4 (806.5‒2477.5)	1538.7 ± 396.4 (742.2‒2712.6)	1588.0 ± 413.0 (764.7‒3025.5)	0.0004	< 0.0001	0.0208	+ 5.3	+ 8.7	+ 3.2
Right upper lobe (Expiratory)	537.7 ± 151.4 (236.2‒911.2)	603.4 ± 156.1 (299.4‒985.8)	611.8 ± 160.8 (306.3‒1066.9)	< 0.0001	< 0.0001	0.1925	+ 12.2	+ 13.8	+ 1.4
Right middle lobe (Expiratory)	282.7 ± 80.3 (150.2‒512.7)	236.7 ± 73.5 (83.0‒432.3)	242.7 ± 77.1 (121.8‒497.1)	< 0.0001	< 0.0001	0.1194	− 16.3	− 14.1	+ 2.5
Right lower lobe (Expiratory)	640.2 ± 178.4 (309.9‒1203.6)	698.6 ± 213.7 (287.6‒1294.6)	733.5 ± 216.1 (293.0‒1461.4)	< 0.0001	< 0.0001	0.0036	+ 9.1	+ 14.6	+ 5.0
Left lung (Expiratory)	1227.4 ± 333.9 (636.2‒2063.5)	1321.1 ± 368.6 (571.6‒2275.1)	1355.6 ± 370.6 (616.1‒2564.0)	< 0.0001	< 0.0001	0.0652	+ 7.6	+ 10.4	+ 2.6
Left upper lobe (Expiratory)	684.6 ± 186.2 (339.7‒1251.4)	724.7 ± 186.7 (359.1‒1237.2)	733.0 ± 191.2 (356.5‒1434.7)	< 0.0001	< 0.0001	0.3584	+ 5.9	+ 7.1	+ 1.1
Left lower lobe (Expiratory)	542.9 ± 168.1 (223.8‒1050.2)	596.4 ± 202.3 (212.5‒1190.6)	622.6 ± 199.6 (189.9‒1137.2)	< 0.0001	< 0.0001	0.0108	+ 9.9	+ 14.7	+ 4.4

P < 0.0167 was considered to be statistically significant, with Bonferroni correction for multiple comparisons.

### Proportional volumes of each lung and each lobe relative to the total lung volume in supine, standing, and sitting positions

In the inspiratory CT, the proportional volumes of the bilateral upper lobes and right middle lobe relative to the total lung volume were significantly lower in the standing and sitting positions than in the supine position, whereas the proportional volumes of the bilateral lower lobes were significantly higher in the standing position than in the supine position (all *P* < 0.001) (Table 3).

**Table 3:** Proportional volumes of each lung and each lobe relative to the total lung volume as determined using CT in supine, standing, and sitting positions (100 volunteers).

	Proportional volumes on CT Average ± SD (Range)			*P* value		
	Supine (%)	Standing (%)	Sitting (%)	Supine vs. standing	Supine vs. sitting	Standing vs. sitting
Total (Bilateral) lung (Inspiratory)	100.0 ± 0.0 (100.0‒100.0)	100.0 ± 0.0 (100.0‒100.0)	100.0 ± 0.0 (100.0‒100.0)	‒	‒	‒
Right lung (Inspiratory)	53.5 ± 1.8 (47.9‒60.8)	53.1 ± 1.5 (49.2‒59.6)	53.3 ± 1.5 (49.2‒59.9)	< 0.0001	0.0905	0.0012
Right upper lobe (Inspiratory)	18.5 ± 2.6 (12.2‒27.4)	18.2 ± 2.3 (12.0‒27.4)	18.2 ± 2.4 (12.3‒27.0)	0.0009	0.0007	0.9670
Right middle lobe (Inspiratory)	9.0 ± 1.7 (4.6‒13.6)	8.2 ± 1.5 (4.4‒12.6)	8.2 ± 1.5 (4.2‒13.0)	< 0.0001	< 0.0001	0.5524
Right lower lobe (Inspiratory)	26.0 ± 2.6 (15.4‒33.3)	26.7 ± 2.2 (18.7‒33.1)	26.9 ± 2.2 (19.1‒33.7)	< 0.0001	< 0.0001	0.0045
Left lung (Inspiratory)	46.5 ± 1.8 (39.2‒52.1)	46.9 ± 1.5 (40.4‒50.8)	46.7 ± 1.5 (40.1‒50.8)	0.0001	0.0901	0.0012
Left upper lobe (Inspiratory)	23.7 ± 2.1 (18.8‒32.2)	23.0 ± 2.0 (17.6‒29.0)	23.0 ± 2.0 (17.7‒29.2)	< 0.0001	< 0.0001	0.5201
Left lower lobe (Inspiratory)	22.8 ± 2.6 (14.3‒28.3)	23.9 ± 2.4 (15.3‒28.3)	23.7 ± 2.4 (15.3‒28.6)	< 0.0001	< 0.0001	0.0133
Total (Bilateral) lung (Expiratory)	100.0 ± 0.0 (100.0‒100.0)	100.0 ± 0.0 (100.0‒100.0)	100.0 ± 0.0 (100.0‒100.0)	‒	‒	‒
Right lung (Expiratory)	54.5 ± 2.0 (47.9‒59.8)	54.0 ± 1.8 (49.9‒59.6)	54.0 ± 1.6 (50.1‒58.8)	0.0003	0.0025	0.3807
Right upper lobe (Expiratory)	20.0 ± 2.5 (13.5‒28.4)	21.3 ± 2.8 (15.8‒28.3)	21.0 ± 2.5 (14.7‒26.8)	< 0.0001	< 0.0001	0.0030
Right middle lobe (Expiratory)	10.7 ± 2.2 (5.5‒16.2)	8.3 ± 1.7 (4.4‒13.5)	8.3 ± 1.6 (4.2‒12.8)	< 0.0001	< 0.0001	0.1194
Right lower lobe (Expiratory)	23.8 ± 2.9 (15.6‒32.4)	24.3 ± 2.6 (16.9‒30.8)	24.8 ± 2.5 (16.7‒30.7)	0.0029	< 0.0001	< 0.0001
Left lung (Expiratory)	45.5 ± 2.0 (40.2‒52.1)	46.0 ± 1.8 (40.4‒50.1)	46.0 ± 1.6 (41.2‒49.9)	0.0003	0.0025	0.3815
Left upper lobe (Expiratory)	25.5 ± 2.4 (20.4‒33.3)	25.5 ± 2.3 (20.1‒33.1)	25.0 ± 2.2 (19.7‒31.8)	0.8679	0.0042	< 0.0001
Left lower lobe (Expiratory)	20.0 ± 2.5 (13.4‒25.5)	20.5 ± 2.7 (13.6‒26.1)	20.9 ± 2.5 (13.4‒26.7)	0.0034	< 0.0001	0.0014

P < 0.0167 was considered to be statistically significant, with Bonferroni correction for multiple comparisons.

### Lung and lobe volume changes from expiration to inspiration on CT in supine, standing, and sitting positions

Volume changes of the total lung, right lung, left lung, right middle lobe, and bilateral lower lobe from expiration to inspiration in the standing and sitting positions were significantly higher than those in the supine position, whereas the bilateral upper lobe volume changes from expiration to inspiration were similar in the standing, sitting, and supine positions (Table 4). No significant differences were found in the all lung and lobe volume changes from expiration to inspiration between the standing and sitting positions (Table 4).

**Table 4:** Lung and lobe volume changes from expiration to inspiration as determined using CT in the supine, standing, and sitting positions (100 volunteers).

	Volume change from expiration to inspiration on CT Average ± SD (Range)			*P* value		
	Supine position (mL)	Standing position (mL)	Sitting position (mL)	Supine vs. standing	Supine vs. sitting	Standing vs. sitting
Total (Bilateral) lung	1598.2 ± 843.7 (117.4‒3972.5)	1822.9 ± 806.9 (535.0‒4259.7)	1769.9 ± 827.0 (48.9‒4181.3)	0.0002	0.0038	0.3217
Right lung	825.8 ± 428.0 (47.9‒1969.9)	942.8 ± 422.7 (292.4‒2232.9)	920.3 ± 430.6 (31.3‒2144.3)	0.0002	0.0020	0.4290
Right upper lobe	248.4 ± 137.3 (− 39.3‒699.6)	244.4 ± 114.6 (19.0‒591.0)	241.0 ± 117.4 (7.1‒632.1)	0.6576	0.4219	0.6656
Right middle lobe	100.3 ± 56.9 (1.9‒265.0)	145.9 ± 80.1 (− 48.8‒375.5)	143.9 ± 80.5 (− 22.9‒409.5)	< 0.0001	< 0.0001	0.6929
Right lower lobe	477.1 ± 250.3 (20.9‒1065.8)	552.5 ± 250.1 (169.0‒1476.4)	535.4 ± 254.0 (− 0.8‒1281.0)	0.0001	0.0014	0.3060
Left lung	772.4 ± 418.3 (2.4‒2002.6)	880.0 ± 387.2 (242.6‒2104.4)	849.6 ± 399.3 (17.4‒2036.9)	0.0002	0.0079	0.2273
Left upper lobe	324.8 ± 180.1 (− 75.5‒891.4)	349.2 ± 164.2 (100.4‒825.6)	345.3 ± 176.2 (0.3‒857.7)	0.0401	0.1049	0.7287
Left lower lobe	447.7 ± 249.6 (− 0.4‒1132.9)	530.9 ± 235.7 (142.2‒1278.8)	504.2 ± 237.8 (3.6‒1179.2)	< 0.0001	0.0010	0.0730

P < 0.0167 was considered to be statistically significant, with Bonferroni correction for multiple comparisons.

### Ratio of inspiratory lung/lobe volume to expiratory lung/lobe volume in supine, standing, and sitting positions

Differences in the ratio of inspiratory volume to expiratory volume in the total lung, right lung, right upper lobe, right lower lobe, left lung, left upper lobe, and left lower lobe between the supine and standing/sitting positions were less than 0.13, whereas the difference in the ratio of inspiratory volume to expiratory volume in the right middle lobe between the supine and standing/sitting positions were more than 0.29 (both P < 0.0001) (Table 5).

**Table 5:** Ratio of inspiratory lung/lobe volume to expiratory lung/lobe volume as determined using CT in the supine, standing, and sitting positions (100 volunteers).

	Ratio of inspiratory volume to expiratory volume on CT Average ± SD (Range)			*P* value		
	Supine position	Standing position	Sitting position	Supine vs. standing	Supine vs. sitting	Standing vs. sitting
Total (Bilateral) lung	1.630 ± 0.360 (1.035‒2.731)	1.693 ± 0.377 (1.209‒2.972)	1.652 ± 0.370 (1.017‒2.862)	0.0436	0.4693	0.1593
Right lung	1.598 ± 0.335 (1.039‒2.574)	1.665 ± 0.368 (1.210‒2.930)	1.629 ± 0.359 (1.020‒2.769)	0.0189	0.2660	0.1964
Right upper lobe	1.502 ± 0.318 (0.957‒2.707)	1.431 ± 0.229 (1.019‒2.132)	1.421 ± 0.238 (1.013‒2.477)	0.0034	0.0008	0.5751
Right middle lobe	1.364 ± 0.201 (1.007‒2.086)	1.688 ± 0.480 (0.873‒3.651)	1.660 ± 0.463 (0.883‒3.551)	< 0.0001	< 0.0001	0.4527
Right lower lobe	1.795 ± 0.447 (1.040‒3.009)	1.886 ± 0.524 (1.251‒3.753)	1.809 ± 0.479 (0.999‒3.253)	0.0275	0.7292	0.0527
Left lung	1.671 ± 0.396 (1.002‒2.941)	1.727 ± 0.394 (1.208‒3.021)	1.680 ± 0.387 (1.013‒2.981)	0.1075	0.7824	0.1241
Left upper lobe	1.514 ± 0.333 (0.940‒2.848)	1.520 ± 0.295 (1.097‒2.539)	1.511 ± 0.322 (1.000‒2.962)	0.8120	0.9258	0.7109
Left lower lobe	1.880 ± 0.506 (0.999‒3.228)	2.003 ± 0.567 (1.252‒3.794)	1.896 ± 0.510 (1.005‒3.338)	0.0116	0.7225	0.0122

P < 0.0167 was considered to be statistically significant, with Bonferroni correction for multiple comparisons.

### Agreements between volumes on CT in supine, standing, and sitting positions and measurements on PFT

Intraclass-correlation coefficients between the total lung volumes on inspiratory CT in the supine/standing/sitting positions and the total lung capacity on PFT were 0.831 (95% confidence interval [CI], 0.759‒0.883)/0.927 (95% CI, 0.893‒0.950)/0.946 (95% CI, 0.920‒0.963), respectively. Intraclass-correlation coefficients between the total lung volumes on expiratory CT in the supine/standing/sitting positions and the functional residual capacity on PFT were 0.834 (95% CI, 0.763‒0.885)/0.848 (95% CI, 0.782‒0.895)/0.811 (95% CI, 0.732‒0.869), respectively. The intraclass-correlation coefficients between the total lung volume changes from expiration to inspiration on CT in the supine/standing/sitting positions and the inspiratory capacity (total lung capacity minus functional residual capacity) on PFT were 0.508 (95% CI, 0.347‒0.640)/0.601 (95% CI, 0.460‒0.713)/0.625 (95% CI, 0.490‒0.731), respectively.

### Associations between volumes on CT in supine, standing, and sitting positions and measurements on PFT

**Figure 3:**
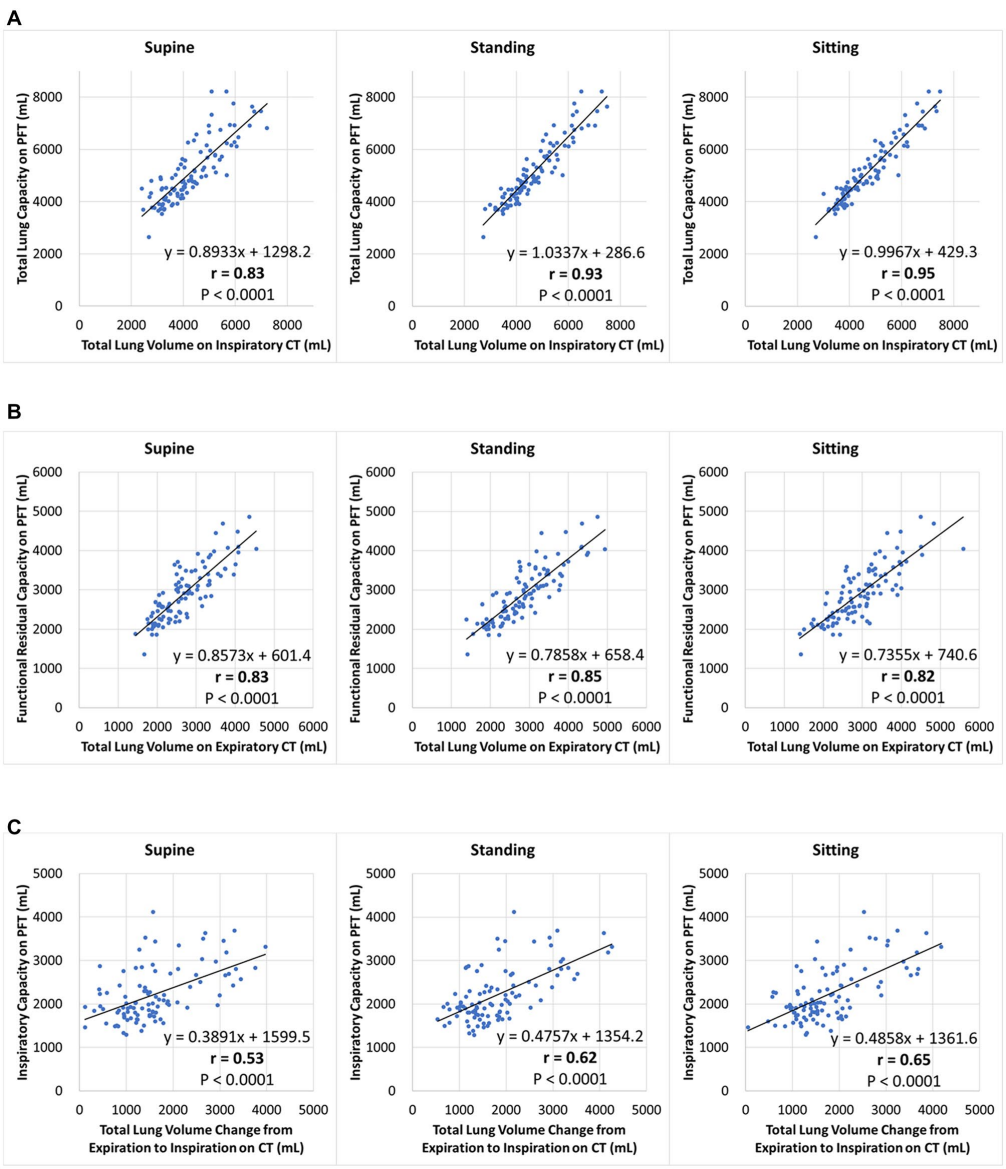
Association between total lung volumes on inspiratory CT in the supine/standing/sitting positions and the total lung capacity on PFT (**A**), association between total lung volumes on expiratory CT in the supine/standing/sitting positions and the functional residual capacity on PFT (**B**), and association between total lung volume changes from expiration to inspiration on CT in the supine/standing/sitting positions and the inspiratory capacity on PFT (**C**), using Pearson correlation coefficient test. (**A**–**C**) Lines show estimated regression.

The correlation coefficient *r* comparing the total lung volumes on inspiratory CT in the supine/standing/sitting positions and the total lung capacity on PFT were, 0.83 (95% CI, 0.76‒0.88)/0.93 (95% CI, 0.90‒0.95)/0.95 (95% CI, 0.92‒0.96), respectively (Fig. 3A). The *r* comparing the total lung volumes on expiratory CT in the supine/standing/sitting positions and the functional residual capacity on PFT were 0.83 (95% CI, 0.76‒0.89)/0.85 (95% CI, 0.78‒0.90)/0.82 (95% CI, 0.73‒0.87), respectively (Fig. 3B). The *r* comparing the total lung volume changes from expiration to inspiration on CT in the supine/standing/sitting positions and the inspiratory capacity on PFT were 0.53 (95% CI, 0.38‒0.66)/0.62 (95% CI, 0.49‒0.73)/0.65 (95% CI, 0.52‒0.75), respectively (Fig. 3C).

### Interobserver and intraobserver agreements

Interobserver and intraobserver agreements (intraclass-correlation coefficients) for measuring CT-based lung and lobe volumes in the supine, standing, and sitting positions were substantial for all measurements (0.998–1.000) (Table 6).

**Table 6:** Interobserver and intraobserver agreements (intraclass-correlation coefficients) for measuring inspiratory and expiratory lung and lobe volumes as determined using CT in the supine, standing, and sitting positions (the first 20 of 100 volunteers).

	Interobserver agreements for measuring Lung and lobe volumes (95% confidence interval)			Intraobserver agreements for measuring Lung and lobe volumes (95% confidence interval)		
	Supine	Standing	Sitting	Supine	Standing	Sitting
Total (Bilateral) lung (Inspiratory)	1.000 (1.000‒1.000)	1.000 (1.000‒1.000)	1.000 (1.000‒1.000)	1.000 (1.000‒1.000)	1.000 (1.000‒1.000)	1.000 (1.000‒1.000)
Total (Bilateral) lung (Expiratory)	1.000 (1.000‒1.000)	1.000 (1.000‒1.000)	1.000 (1.000‒1.000)	1.000 (1.000‒1.000)	1.000 (1.000‒1.000)	1.000 (1.000‒1.000)
Right lung (Inspiratory)	1.000 (1.000‒1.000)	1.000 (1.000‒1.000)	1.000 (1.000‒1.000)	1.000 (1.000‒1.000)	1.000 (1.000‒1.000)	1.000 (1.000‒1.000)
Right lung (Expiratory)	1.000 (1.000‒1.000)	1.000 (1.000‒1.000)	1.000 (1.000‒1.000)	1.000 (1.000‒1.000)	1.000 (1.000‒1.000)	1.000 (1.000‒1.000)
Right upper lobe (Inspiratory)	1.000 (0.999‒1.000)	1.000 (1.000‒1.000)	1.000 (1.000‒1.000)	1.000 (0.999‒1.000)	1.000 (1.000‒1.000)	1.000 (1.000‒1.000)
Right upper lobe (Expiratory)	1.000 (0.999‒1.000)	1.000 (0.999‒1.000)	1.000 (0.999‒1.000)	1.000 (1.000‒1.000)	1.000 (1.000‒1.000)	1.000 (1.000‒1.000)
Right middle lobe (Inspiratory)	0.999 (0.998‒1.000)	0.999 (0.999‒1.000)	0.998 (0.995‒0.999)	0.999 (0.998‒1.000)	1.000 (0.999‒1.000)	0.999 (0.998‒1.000)
Right middle lobe (Expiratory)	0.999 (0.997‒1.000)	0.998 (0.996‒0.999)	0.998 (0.994‒0.999)	1.000 (0.999‒1.000)	1.000 (0.999‒1.000)	0.999 (0.997‒0.999)
Right lower lobe (Inspiratory)	1.000 (1.000‒1.000)	1.000 (1.000‒1.000)	1.000 (0.999‒1.000)	1.000 (1.000‒1.000)	1.000 (1.000‒1.000)	1.000 (1.000‒1.000)
Right lower lobe (Expiratory)	1.000 (1.000‒1.000)	1.000 (1.000‒1.000)	1.000 (0.999‒1.000)	1.000 (1.000‒1.000)	1.000 (1.000‒1.000)	1.000 (1.000‒1.000)
Left lung (Inspiratory)	1.000 (1.000‒1.000)	1.000 (1.000‒1.000)	1.000 (1.000‒1.000)	1.000 (1.000‒1.000)	1.000 (1.000‒1.000)	1.000 (1.000‒1.000)
Left lung (Expiratory)	1.000 (1.000‒1.000)	1.000 (1.000‒1.000)	1.000 (1.000‒1.000)	1.000 (1.000‒1.000)	1.000 (1.000‒1.000)	1.000 (1.000‒1.000)
Left upper lobe (Inspiratory)	1.000 (1.000‒1.000)	1.000 (1.000‒1.000)	1.000 (0.999‒1.000)	1.000 (1.000‒1.000)	1.000 (1.000‒1.000)	1.000 (1.000‒1.000)
Left upper lobe (Expiratory)	1.000 (0.999‒1.000)	1.000 (0.999‒1.000)	1.000 (0.999‒1.000)	1.000 (1.000‒1.000)	1.000 (1.000‒1.000)	1.000 (1.000‒1.000)
Left lower lobe (Inspiratory)	1.000 (1.000‒1.000)	1.000 (1.000‒1.000)	1.000 (1.000‒1.000)	1.000 (1.000‒1.000)	1.000 (1.000‒1.000)	1.000 (1.000‒1.000)
Left lower lobe (Expiratory)	1.000 (0.999‒1.000)	1.000 (0.999‒1.000)	1.000 (0.999‒1.000)	1.000 (0.999‒1.000)	1.000 (1.000‒1.000)	1.000 (1.000‒1.000)

## Discussion

Our prospective study demonstrated that (1) inspiratory as well as expiratory CT-based volumes of the bilateral upper lobes, bilateral lower lobes, bilateral lungs, and total lungs were significantly higher in the standing and sitting positions than in the supine position, (2) no significant differences were found in the inspiratory right middle lobe volumes among the three positions and the expiratory right middle lobe volume was significantly lower in the standing and sitting positions than in the supine position, and (3) no significant differences were found in the inspiratory volumes of the total lung, right lung, left lung, and all lobes between the standing position and the sitting position. These data could have an impact on preoperative CT volumetry of the lung in patients with lung cancer (before lobectomy) for the prediction of postoperative residual pulmonary function. In addition, our findings of 100 asymptomatic volunteers could be used as the basis for elucidating undetermined pathological mechanisms, particularly when the symptoms of pulmonary diseases vary between supine and upright (standing and sitting) positions or when they have a predilection for a pulmonary lobe.

We found that the collapse of the right middle lobe in expiratory CT was more apparent in the standing/sitting position than in the supine position. The underlying etiology of nonobstructive right middle lobe syndrome is not yet well understood^20^, but there is a possibility that our data may provide clues to understanding the pathogenic mechanism of nonobstructive right middle lobe syndrome. Regarding inspiratory CT, a previous study reported that the inspiratory right middle lobe volume was similar between the supine and standing positions (with the subjects' arms down)^6^; with regard to the inspiratory volume, our conventional supine CT-based results with subjects’ arms raised (usually performed posture) are consistent with the previously reported results.

Our study also demonstrated that the bilateral upper lobe volume changes from expiration to inspiration were similar among the three positions. This may be explained by the previously reported phenomenon that the lower lungs undergo greater volume changes during breathing than do the upper lungs because of the effects of gravity on lung recoil^6,21–23^.

Our study showed that the total lung volume determined using inspiratory upright CT (in standing and sitting positions) was more similar to and more correlated with the total lung capacity on PFT than that determined in the supine position (intraclass-correlation coefficient, 0.927 and 0.946 vs. 0.831; *r*, 0.93 and 0.95 vs. 0.83). Furthermore, although associations between the total lung volumes on expiratory CT and the functional residual capacity on PFT were similar in the three positions, the total lung volume changes from expiration to inspiration determined using upright CT (in standing and sitting positions) tended to be more correlated with the inspiratory capacity on PFT than that determined using supine CT (*r*, 0.62 and 0.65 vs. 0.53). This may be because the PFT was performed in the sitting (upright) position, and the direction of the thorax in the PFT was more similar to that in upright CT than that in conventional supine CT^6^. Previous studies have reported that functional residual capacity on PFT was higher in the standing^24^ and sitting^25^ than in supine position among healthy subjects^26^. Our results of the total lung capacity on expiratory CT were consistent with those of these previous studies. As of June 23, 2020, there were more than 9.1 million confirmed cases of coronavirus disease 19 (COVID-19) globally^27^. To reduce the spread of COVID-19, many PFT laboratories have been closed or have significantly reduced their testing capacity at the time of writing this manuscript^27^. Considering our results, in addition to the morphological evaluation of the chest, inspiratory and expiratory upright CT could be used as an alternative tool to predict lung volumes such as total lung capacity, functional residual capacity, and inspiratory capacity in situations in which PFT cannot be performed, with relatively more accurate predictability compared with conventional supine CT. Nonetheless, in a previous study evaluating only inspiratory lung volume in the supine and standing positions (with the subject’s arms down)^6^, the acquired CT data could not provide a prediction of the functional residual capacity or inspiratory capacity because of the lack of expiratory CT data. In this study, to perform both inspiratory and expiratory CT, we used a tube voltage of 100 kVp and automatic exposure control with a noise index of 24 for a slice thickness of 5 mm (relatively low-radiation-dose protocol) because the low-radiation-dose CT with recent scanner technology or reconstruction methods is reported to be sufficient to detect lung cancers^28^, nodules^29^, and pneumonia including COVID-19 pneumonia^30,31^ and because low radiation dose is reported to be unrelated to the volume or area measurement accuracy^32^, whereas a previous study^6^ evaluating only inspiratory CT in the supine and standing positions used a tube voltage of 120 kVp and a noise index of 15.

Our study had several limitations. First, we included only 100 asymptomatic subjects at a single institution, and further studies in larger populations at multiple centers are required to confirm these preliminary findings. Second, in this study, although the observers independently evaluated the CT images in a randomized and blinded manner, they could recognize, to some extent, the positions of the subjects because of the absence or presence of a CT table. However, the lung and lobe volume measurements were semi-automated by using a commercially available workstation, and thus, the observer bias would have been negligible^6^. Furthermore, the interobserver and intraobserver agreements (intraclass-correlation coefficients) were high (> 0.99) in this study. Third, although all the participants underwent PFTs within two hours of CT examinations, PFTs were not performed simultaneously with the CT, which could have affected the results of the correlation between CT and PFT measurements. However, it was impossible to perform PFT including total lung capacity measurements in the bore of the CT.

## Conclusions

Although the inspiratory and expiratory CT-based volumes of the bilateral upper lobes, bilateral lower lobes, bilateral lungs, and total lungs were significantly greater in the standing and sitting positions (with arms down) than in the supine position (with arms raised), the inspiratory right middle lobe volume did not change significantly in the three positions and the expiratory right middle lobe volume was significantly lower in the standing and sitting positions than in the supine position. The inspiratory volumes of the total lung, right lung, left lung, and all lobes were similar for the standing and sitting positions. The total lung volumes on inspiratory upright CT in the upright position (standing and sitting positions) were more similar to the total lung capacity on PFT than that on conventional CT in the supine position. Although associations between the total lung volumes on expiratory CT and the functional residual capacity on PFT were similar in the three positions, the total lung volume changes from expiration to inspiration determined using upright CT (standing and sitting positions) tended to be more closely correlated with the inspiratory capacity on PFT than volume changes determined using supine CT.

## Data Availability

The datasets generated during and/or analysed during the current study are available from the corresponding author on reasonable request.
